# Co-Opetition Strategies of Superior and Subordinate Hospitals for Integration of Electronic Health Records Within the Medical Consortiums in China Based on Two-Party Evolutionary Game Theory: Mixed Methods Study

**DOI:** 10.2196/70866

**Published:** 2025-08-14

**Authors:** Shenghu Tian, Rong Jiang, Jianfeng Yao, Yu Chen

**Affiliations:** 1 Business School, Yunnan University of Finance and Economics, Kunming, China; 2 Yunnan Provincial Key Laboratory of Service Computing, Kunming, China; 3 Information School, Yunnan University of Finance and Economics, Kunming, China; 4 Continuing Education School, Yunnan University of Finance and Economics, Kunming, China; 5 Management and Economics School, Kunming University of Science and Technology, No. 68 Wenchang Road, Wuhua District, Kunming, 650093, China, 86 13078714962

**Keywords:** electronic health records, EHR, medical consortiums, evolutionary game, co-opetition strategy

## Abstract

**Background:**

Medical consortiums take the integration of electronic health records (EHR) as a breakthrough point and the construction of an integrated medical service system as the ultimate goal. However, their establishment has disrupted the balance between the original medical order and interest patterns. While promoting active cooperation among hospitals, it has also intensified active competition between them.

**Objective:**

This study aimed to explore the internal evolution mechanism of the co-opetition strategies adopted by the superior and subordinate hospitals in the medical consortiums, providing a theoretical foundation and policy reference for achieving EHR integration.

**Methods:**

On the basis of analyzing the structure of the main players in the co-opetition game and their game motivations, we established an evolutionary game model, analyzed the impact mechanism of key parameters, simulated the dynamic evolution process of the co-opetition strategies using MATLAB (MathWorks), and finally proposed actionable policy recommendations.

**Results:**

The results indicate that three factors positively promote EHR integration: (1) EHR complementarity, (2) hospitals’ willingness and ability to use EHR, and (3) the average revenue per unit of EHR. Conversely, the investment cost per unit of resources hinders EHR integration. Neither the original income of hospitals nor the stock of EHR significantly affects the evolution direction of the game system.

**Conclusions:**

Medical consortiums should actively involve all levels and different types of medical institutions, and continuously improve hospitals’ willingness and ability to use EHR through training, assistance, support, and sinking of medical resources, etc. The government should establish a reward and punishment system, optimize the operation and supervision mechanism of medical consortiums, and monitor and punish opportunism behaviors such as “free-riding.” It is also crucial to strengthen the construction of hospital informatization infrastructure and improve the technical, content, and sharing standards for EHR construction. In addition, designing reward and punishment mechanisms as well as cost accounting based on “unit EHR resources” is also of great significance for promoting the EHR integration.

## Introduction

In the traditional health care delivery system, medical institutions were fragmented and scattered, and hospitals competed for access to patients in order to achieve the goal of maximizing their own interests, but active cooperation in medical care was basically nonexistent. To address this fragmentation and scattering of health care services and achieve an integrated health care delivery pattern of services, responsibilities, benefits, and management [1]. Medical consortium building has become a major strategic choice for countries around the world. Medical consortiums are a type of health care alliance that has gained attention in China in recent years. In China, medical consortiums, a form of health care alliance that has gained significant attention in recent years, primarily manifest in 4 organizational models: urban medical groups, county-level medical communities, cross-regional specialty alliances, and telemedicine collaboration networks [2]. Medical consortiums typically use electronic health records (EHR) integration as a foundational strategy to realize the objectives of “primary diagnosis at grassroots institutions, two-way referrals, tiered treatment for acute and chronic cases, and coordinated care across different levels of healthcare providers,” and ultimately building an integrated medical service system. However, the development of medical consortiums has broken the balance of the original medical order and interest pattern, and has intensified active competition among hospitals while promoting active cooperation among them. Consequently, in the process of promoting the EHR integration within medical consortiums, hospitals inevitably adopt co-opetition strategies to safeguard and maximize their own interests.

Brandenburger and Nalebuff [3], who developed the concept of co-opetition, believe that the organization’s operating activities are not all zero-sum games, but also win-win nonzero-sum games, and that the strategy to achieve this win-win outcome is “co-opetition strategy.” In fact, competition and cooperation are the driving force for sustainable economic and social development [4]. The social value of medical consortiums predominantly stems from the co-opetition relationship among member. Regarding EHR integration, hospitals at all levels are characterized by imperfect rationality and imperfect information. Evolutionary game can be used to reveal the competition and cooperation relationship between players and the law of their strategy selection. Existing studies have primarily examined: (1) individual hospitals’ data-sharing willingness [5], (2) access control models [6], and (3) the game between the government and the hospital [7]. However, these studies have inadequately analyzed the competition and cooperation relationship between the superior and subordinate hospitals in depth from the perspective of medical consortiums, and also failed to reveal the evolution mechanism of competition and cooperation strategies choices, which has limited the practical reference value of research results for policy optimization.

As far as the related research is concerned, evolutionary game theory is favored because its finite rationality assumption is more realistic, and has wide applications in the fields of multivariate governance, organizational synergy, and information behavior [8]. For instance, evolutionary game models have been used to analyze interest equilibria in open data ecosystems [9], revealing the impact of the changing psychology of individuals on group behavior [10]. In health care applications, evolutionary game theory has been principally applied to investigate: (1) privacy disclosure behaviors in “online health communities” [11], (2) quality assurance strategies for emergency medical supplies [12], (3) selection behaviors regarding internet hospitals [13], (4) models of epidemic information dissemination [14], and (5) hierarchical diagnosis and treatment strategy and 2-way referral mechanism [15,16]. The related research reasonably explains the evolution mechanism of relevant strategy selection behavior, and establishes a solid methodological foundation for our study.

China’s EHR development has progressed through 3 evolutionary phases, namely independent construction, system integration, and shared usage. In the initial phase, EHR was built separately by each hospital, realizing a breakthrough from scratch, and the construction is effective and widely applied within the hospital. The EHR integration is the premise and foundation of crossinstitutional “sharing and utilization,” which is the key link in releasing the EHR application, and also the bottleneck in the EHR development at present. There exists an urgent need to elucidate the co-opetition strategies between superior and subordinate hospitals facilitating EHR integration within medical consortiums, thereby establishing a theoretical foundation for policy optimization. Previous studies suggested that evolutionary games provide a valuable framework for elucidating the emergence and maintenance of cooperative behavior [17]. Accordingly, this study uses evolutionary game theory to uncover the intrinsic mechanisms governing co-opetition strategy evolution between superior and subordinate hospitals during EHR integration within medical consortiums. The study comprises three key components: (1) from the perspective of the evolution of co-opetition strategies, analyzing the structure of competition and cooperation game subjects and their game motivations; (2) establishing a game model for the evolution of the competition of the superior and subordinate hospitals; and (3) simulating the dynamic evolution process of the co-opetition strategy using MATLAB (MathWorks). This study bridges the previous gap of focusing only on superficial policy recommendations and neglecting to reveal the game mechanism of multiple subjects behind the policies. The findings provide both theoretical foundations and policy-relevant insights for achieving EHR integration objectives.

## Methods

### Research Design

This study followed the research approach of “basic assumptions - model construction - result analysis - numerical simulation.” First, for the convenience of modeling and analysis, combined with the actual situation of EHR integration in China, the superior hospital A and its subordinate hospital B were randomly set as research objects within the medical consortiums. Referring to related studies on cooperative competitive games in the big data innovation alliance and information ecosystem [18,19], the basic assumptions were put forward from 6 aspects: game players, co-opetition relationships, value-added benefits, synergistic effects, co-operation costs, and reward-punishment system. Second, an evolutionary game model of the competitive and cooperative behaviors between the superior and subordinate hospitals was constructed. Then, we analyzed the influence mechanisms of key factors on the selection of competition and cooperation strategies, and MATLAB was used to conduct simulation experiments on the model operation. Finally, targeted policy recommendations were proposed to highlight the practical reference value of the research conclusions.

### Underlying Assumptions

#### Game Players and Co-Opetition Relationships

In China, medical consortiums consist of dozens or even hundreds of hospitals at different levels and scales, including tertiary hospitals, secondary hospitals, county hospitals, community hospitals, and rural hospitals. These hospitals belong to different levels of administrative divisions. In the existing medical order designed by the “hierarchical diagnosis and treatment” system, these hospitals present an unstructured hierarchical relationship with each other. When a patient is transferred from a county hospital to a tertiary hospital for treatment, the county hospital becomes a subordinate hospital to the tertiary hospital; when a patient is transferred from a community hospital to a county hospital for treatment, the county hospital is again a superior hospital to the community hospital. Obviously, “superior and subordinate hospitals” is a relative concept, but it can accurately portray the main structure of the co-opetition game among hospitals at each level in medical consortiums. Therefore, we classify the players participating in the co-opetition evolutionary game into superior hospitals and subordinate hospitals.

The superior and subordinate hospitals have not only formed a cooperative relationship based on policy requirements and integration goals but also formed a competitive relationship due to risk avoidance and interest maximization. They continuously create and add value through both competition and cooperation. In summary, the co-opetition relationship between superior and subordinate hospitals manifests in the following four situations [20]: (1) Creation of integrated medical value (cooperation): improves health care service quality and alleviates the social contradictions of difficult and expensive access to health care; (2) pursuit of patient volume (competition): the number of patients directly determines the hospital’s financial gains; (3) policy and contractual regulation (cooperation): hospitals must comply with the requirements of the hierarchical diagnosis and treatment system, share EHR and implement referral regulations; and (4) opportunistic behavior (competition): may include cost-input bargaining, income concealment, inaction, and “free-riding” situations.

Hospitals are profit-seeking entities that aim to maximize their own interests in the co-opetition process [21]. Hospitals compare their benefits and strategies with reference hospitals and adjust their strategies accordingly, resulting in various game behaviors. Influenced by changes in external factors and constrained by imperfect rationality and incomplete information, hospitals can only continuously adjust their strategies according to a certain probability to maximize income, which forms an evolutionary game. Assume both hospitals *A* and *B* have 2 optional strategies in EHR integration, namely competition and cooperation. The probability of hospital *A* choosing cooperation is *x*, then the probability of hospital *A* choosing competition is 1-*x*; similarly, hospital *B*’s g cooperation probability is *y*, making its competition probability 1-*y*. where x,y∈[0,1] and *x *and *y* are both functions of time *t*.

#### Value-Added Benefits and Synergistic Effects

The EHR integration can easily realize “two-way referral,” enabling *A* and *B* to gain value-added benefits. The value-added benefits include not only economic benefits, but also noneconomic benefits, such as social reputation, brand image, collective honor, hospital responsibility, superior praise, promotion, and public praise. The benefits that hospitals were able to obtain before EHR integration can be referred to as the original benefits and are denoted by $R_{i}$. Whether EHR integration can generate value-added and the magnitude of the value-added depends on the hospital’s willingness to use EHR $\rho_{i})({0\leq \rho }_{i}\leq 1,i=A,B)$, the ability to use EHR ${(\mu }_{i})(0\leq \mu_{i}\leq 1,i=A,B)$, and the number of the other’s health records shared $\left(r_{i})({0\leq \mu }_{i},i=A,B\right)$. It is assumed that the average benefit per unit resource is *I* (*I*≥0).

A hospital’s willingness to use EHR is fundamentally determined by the decision makers’ cognition, which includes both the decision makers’ awareness of realizing the social value of the medical consortium and their perceived future expected use. Therefore, a higher $\rho_{i}$ indicates that the decision makers perceive greater value and future utility of EHR integration, while a smaller value of $\rho_{i}$ represents that the decision makers pay more attention to immediate and local interests. The ability to use EHR reflects the level of medical care in the hospital, and it includes both the ability of physicians to use the EHR and the medical equipment conditions that the hospital has to use the external EHR. The number of EHRs shared is proportional to the value-added benefit of the other party. The more EHRs shared by the sharing party, the greater value-added benefit the user may gain. Therefore, the value-added benefits received by hospital *A* and *B* are $\rho_{A}\mu_{A}r_{B}I$ and $\rho_{B}\mu_{B}r_{A}I$, respectively.

Usefulness, the ultimate goal of the game, refers to the material or spiritual benefits players obtain through the game [22]. Synergistic effects refer to the enhanced profitability from mutual EHR sharing among hospitals, representing the core objective of medical consortia in promoting EHR integration When both *A* and *B* choose the “cooperation” strategy, synergy will occur, which depends on the hospital’s willingness to use EHR ($\rho_{i}$), the ability to use EHR ($\mu_{i}$) and the EHR resources complementarity (β). Then the synergy benefits of *A* and *B* are ${\beta \rho_{A}\mu_{A}r}_{A}I$ and ${\beta {\rho_{B}\mu }_{B}r}_{B}I$ respectively.

#### Cooperation Cost and Reward-Punishment Mechanism

EHR integration can impose additional costs on hospitals, such as direct costs (IT infrastructure modification and file formatting), risk costs (data privacy/security issues), opportunity costs (potential patient attrition), and indirect costs (reduced revenue from test information reuse) [23]. Existing research suggests that high fines can not only prevent free riding but they also demotivate some of the most generous altruists [24]. To promote the smooth integration of EHRs in medical consortiums, local governments often establish reward and punishment systems to achieve cost compensation while reducing opportunism. The reward and punishment system are the most easily regulated and more frequently adjusted factor, and its changes are more likely to cause changes in the competing strategies of the superior and subordinate hospitals, which in turn affects the evolutionary game direction. Assuming that the average input cost in terms of human, financial, and other resources required to integrate unit resources is *C*, the input costs of *A* and *B* are $r_{A}C$ and $r_{B}C$, respectively. When hospitals actively share and use EHRs for integration purposes, the government grants a unit resource subsidy of *H*, otherwise a unit resource penalty of *H* is granted.

According to the game motives of the players and the above assumptions, the co-opetition relationship between the 2 sides of the game can be portrayed by the following scenarios. First, when both players choose “cooperation,” the benefits obtained by both players include the original benefit ($R_{i}$), value-added benefit ($r_{i}I$), and government incentive ($r_{i}H$). The synergy benefits generated by *A* and *B* are ${\beta \rho_{A}\mu_{A}r}_{A}I$ and${{\rho_{B}\mu }_{B}r}_{B}I$. Also considering the costs $r_{A}C$ and $r_{B}C$ due to the inputs in the cooperation, we can derive the benefit functions of *A* and *B* as ${R_{A}+\rho }_{A}\mu_{A}{(r}_{B}I+{\beta r}_{A}I)+r_{A}H-r_{A}C$ and ${R_{B}+\rho }_{B}\mu_{B}(r_{A}I+{\beta r}_{B}I)+r_{B}H-r_{B}C$. Second, when 1 player chooses “cooperation” and the other chooses “competition.” When *A* chooses “cooperation” and *B* chooses “competition,” *A*’s benefits include the original benefit *R_A_* and financial subsidy $r_{A}H$, and *A* incurs investment costs $r_{A}C$ due to the choice of cooperation. At this time, *A* cannot use *B*’s EHR, and *A*’s value-added benefits are 0. Therefore, *A*’s benefit function is $R_{A}+r_{A}H-r_{A}C$. The benefit of *B* includes both the original benefit *R_B_* and the value-added benefit $R_{B}+\rho_{B}\mu_{B}r_{A}I-r_{B}H$ from free-riding. At this point, *B* has no input cost but receives a penalty $r_{B}H$ from the government. Therefore, the benefit function of *B* is $R_{B}+\rho_{B}\mu_{B}r_{A}I-r_{B}H$. Similarly, when *B* chooses “cooperation” and *A* chooses “competition,” the benefit function of *A* is $R_{A}+\rho_{A}\mu_{A}r_{B}I-r_{A}H$, and the benefit function of *B* is $R_{B}+r_{B}H-r_{B}C$. Finally, when both players choose “competition,” both players gain only the original benefits *R_A_* and *R_B_*, and the penalties for both players are $r_{A}H$ and $r_{B}H$.

When 1 player chooses “cooperation” and the other chooses “competition.” When *A* chooses “cooperation” and *B* chooses “competition,” *A*’s benefits include the original benefit *R_A_* and financial subsidy $r_{A}H$, and *A* incurs investment costs $r_{A}C$ due to the choice of cooperation. At this time, *A* cannot use *B*’s EHR, and *A*’s value-added benefits are 0. Therefore, *A*’s benefit function is $R_{A}+r_{A}H-r_{A}C$. The benefit of *B* includes both the original benefit *R_B_* and the value-added benefit $R_{B}+\rho_{B}\mu_{B}r_{A}I-r_{B}H$ from free-riding. At this point, *B* has no input cost but receives a penalty $r_{B}H$ from the government. Therefore, the benefit function of *B* is $R_{B}+\rho_{B}\mu_{B}r_{A}I-r_{B}H$. Similarly, when *B* chooses “cooperation” and *A* chooses “competition,” the benefit function of *A* is $R_{A}+\rho_{A}\mu_{A}r_{B}I-r_{A}H$, and the benefit function of *B* is$R_{B}+r_{B}H-r_{B}C$.

When both players choose “competition.” Both players gain only the original benefits *R_A_* and *R_B_*, and the penalties for both players are $r_{A}H$ and $r_{B}H$.

### Model Construction

As a result, an evolutionary game payment matrix can be constructed for hospitals *A* and *B*, as shown in Table 1.

**Table 1.** Evolutionary game payment matrix.

Players and optional strategies	Subordinate hospital *B*	
Superior hospital *A*	Cooperation (*y*)	Competition (1-*y*)
Cooperation (*x*)	${R_{A}+\rho }_{A}\mu_{A}{(r}_{B}I+{\beta r}_{A}I)+r_{A}H-r_{A}C$	$R_{A}+r_{A}H-r_{A}C$
	${R_{B}+\rho }_{B}\mu_{B}(r_{A}I+{\beta r}_{B}I)+r_{B}H-r_{B}C$	$R_{B}+\rho_{B}\mu_{B}r_{A}I-r_{B}H$
Competition (1-*x*)	$R_{A}+\rho_{A}\mu_{A}r_{B}I-r_{A}H$	$R_{A}-r_{A}H$
	$R_{B}+r_{B}H-r_{B}C$	$R_{B}-r_{B}H$

According to Table 1, the expected benefit $E_{A1}$ of the cooperation strategy chosen by *A* is:


(1) $$E_{A1}=y\left[R_{A}+\rho_{A}\mu_{A}\left(r_{B}I+\beta r_{A}I\right)+r_{A}H-r_{A}C\right]+\left(1-y\right)\left(R_{A}+r_{A}H-r_{A}C\right)$$

The expected benefit of the competitive strategy chosen by *A* is $E_{A2}$:


(2) $$E_{A2}=y\left[R_{A}+\rho_{A}\mu_{A}r_{B}I-r_{A}H\right]+\left(1-y\right)\left(R_{A}-r_{A}H\right)$$

Then the average expected benefit ${\overset{-}{E}}_{A}$ of superior hospital *A* is:


(3) $${\overset{-}{E}}_{A}=xE_{A1}+\left(1-x\right)E_{A2}=R_{A}+xr_{A}\left(2H-C\right)+y\left(x\beta r_{A}+r_{B}\right)\rho_{A}\mu_{A}I-r_{A}H$$

Therefore, the replication dynamic equation of the cooperation strategy chosen by the superior hospital *A* can be derived as follows:


$$F(x)=\frac{dx}{dt}=x(E_{A1}-{\bar{E}}_{A})$$

(4)$=x\left(1-x\right)\left[r_{A}\left(2H-C\right)+y\beta \rho_{A}\mu_{A}r_{A}I\right]$

Similarly, the expected benefit $E_{B1}$ of the cooperation strategy chosen by *B* is:


(5) $$E_{B1}=x\left[R_{B}+\rho_{B}\mu_{B}\left(r_{A}I+\beta r_{B}I\right)+r_{B}H-r_{B}C\right]+\left(1-x\right)\left(R_{B}+r_{B}H-r_{B}C\right)$$

The expected benefit of the competitive strategy chosen by *B* is $E_{B2}$：


(6) $$E_{B2}=x\left[R_{B}+\rho_{B}\mu_{B}r_{A}I-r_{B}H\right]+\left(1-x\right)\left(R_{B}-r_{B}H\right)$$

Then the average expected benefit ${\overset{-}{E}}_{B}$ of subordinate hospital *B* is:


(7) $${\overset{-}{E}}_{B}=yE_{B1}+\left(1-y\right)E_{B2}=R_{B}+yr_{B}\left(2H-C\right)+x\left(r_{A}+y\beta r_{B}\right)\rho_{B}\mu_{B}I-r_{B}H$$

Therefore, the replication dynamic equation of the cooperation strategy chosen by the subordinate hospital *B* can be derived as follows:


(8) $$F\left(y\right)=\frac{dy}{dt}=y\left(E_{B1}-{\overset{-}{E}}_{B}\right)=y\left(1-y\right)\left[r_{B}\left(2H-C\right)+x\beta \rho_{B}\mu_{B}r_{B}I\right]$$

### Ethical Considerations

No formal ethical approvement was necessary for the study according to the China Science and Technology Ethics Committee.

## Results

### Stability of Equilibrium Points and Their Evolutionary Path Analysis

In the evolutionary game process, hospitals *A* and *B* participate in the game through repeated trial and error, learning and imitation, constantly adjusting their own strategies, so that the dynamic system will eventually reach a stable state. According to the stability theorem of differential equations, let:


(9) $$\left\{\begin{array}{c} F(x)=\frac{dx}{dt}=0\\ F(y)=\frac{dy}{dt}=0 \end{array}\right.$$

By solving equation (9), 5 local equilibrium points of the dynamic evolutionary game system can be obtained, which are *O* (0,0), *F* (0,1), *G* (1,0), *K* (1,1)*, and T* ($\frac{C-2H}{\beta \rho_{B}\mu_{B}I}$,$\;\frac{C-2H}{\beta \rho_{A}\mu_{A}I}$). These 5 equilibrium points together constitute the boundary $\left\{\left(x,y\right)|0\leq x\leq 1;0\leq y\leq 1\right\}$of the evolutionary game system solution domain of superior and subordinate hospitals in EHR integration.

The Jacobi matrix can be obtained by solving the partial derivatives of (4) and (8) with respect to x and y, respectively.


$$J=\left[\begin{array}{cc} \frac{\partial F\left(x\right)}{\partial x} & \frac{\partial F\left(x\right)}{\partial y}\\ \frac{\partial F\left(y\right)}{\partial y} & \frac{\partial F\left(y\right)}{\partial x} \end{array}\right]$$

(10) $$=\left[\begin{array}{cc} \left(1-2x\right)\left[r_{A}\left(2H-C\right)+y\beta \rho_{A}\mu_{A}r_{A}I\right] & x\left(1-x\right)\beta \rho_{A}\mu_{A}r_{A}I\\ y\left(1-y\right)\beta \rho_{B}\mu_{B}r_{B}I & \left(1-2y\right)\left[r_{B}\left(2H-C\right)+x\beta \rho_{B}\mu_{B}r_{B}I\right] \end{array}\right]$$

**Figure 1.**
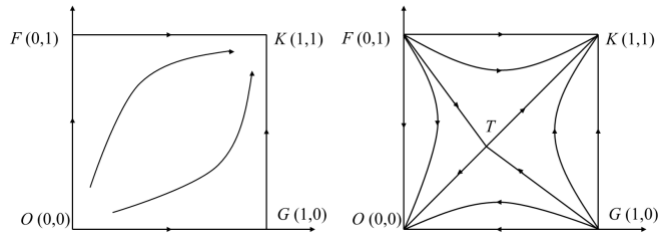
Evolutionary path phase diagram.

For a 2-party behavior dynamic system described by differential equations, whether the above 5 local equilibrium points are evolutionary stable strategies of the dynamic system can be determined by the sign of the det(J) and tr(J) of the Jacobi matrix [8,25]. When 2H>C, the stability of the 5 equilibrium points can be obtained as shown in Table 2. This situation is theoretically valid but difficult to realize in reality. When 2H<C, Since $0<\frac{C-2H}{\beta \rho_{B}\mu_{B}I}<1$ and $0<\frac{C-2H}{\beta \rho_{A}\mu_{A}I}<1$, we can obtain $\beta \rho_{B}\mu_{B}I>C-2H$ and $\beta \rho_{A}\mu_{A}I>C-2H$, and then obtain the stability of the 5 equilibrium points as shown in Table 3 and Figure 1 (2H>C). This situation is more realistic and has a solid practical basis. Therefore, we chose this situation to carry out an in-depth analysis in order to clarify the influence mechanism and influence effect of key factors on the evolutionary game system. The evolutionary path of the dynamic system is shown in Figure 1, where 1 denotes the cooperative strategy and 0 denotes the competitive strategy.

**Table 2.** Equilibrium points of evolutionary game system and their stability (2H>C).

Equilibrium points	Sign of det (J)	Sign of tr (J)	Attribute
O (0,0)	+	+	Unstable point
F (0,1)	−-	NO	Saddle point
G (1,0)	−-	NO	Saddle point
K (1,1)	+	−-	ESS^a^
T (x^*^, y^*^)	−-	0	Saddle point

a ESS: evolutionary stable strategies.

**Table 3.** Equilibrium points and their stability (2H>C).

Equilibrium points	Sign of det (J)	Sign of tr (J)	Attribute
O (0,0)	+	−-	ESS^a^
F (0,1)	+	NO	Unstable point
G (1,0)	+	NO	Unstable point
K (1,1)	+	−-	ESS
T (x^*^, y^*^)	−-	0	Saddle point

a ESS: evolutionary stable strategies.

It can be seen from Table 2 and Figure 1 (2H<C), the evolutionary game system has 2 stable points, *O* (0, 0) and *K* (1, 1). The game strategies corresponding to the stable points are {competition, competition} and {cooperation, cooperation}. *F* (0, 1) and *G* (1, 0) are the unstable points, and their corresponding game strategies are {competition, cooperation} and {cooperation, competition}, respectively. The equilibrium points *T* (x*, y*) is a saddle point located in the plane$\left\{\left(x,y\right)|0\leq x\leq 1;0\leq y\leq 1\right\}$. When the initial state of the dynamic system is within the FTGK region, the evolutionary result will converge to the stability point *K* (1, 1), that is, both the hospitals *A* and *B* choose the cooperation strategy to actively promote the EHR integration. When the initial state of the dynamic system is within the FTGO region, the evolutionary result will converge to the stability point *O* (0, 0), that is, both A and B choose the competition strategy. The final convergence of the evolutionary game system to that stable point depends on the prediction and judgment of both sides of the game on their own ability and the other side’s strategy choice. The willingness and ability of hospitals to use EHR, the number of EHR shared by the other party, and the complementarity of EHR have positive effects on the integration.

### Analysis of the Influence Mechanism of Key Factors

It can be seen from Figure 1 that the size of FTGK area depends on the location of saddle point *T* ($\frac{C-2H}{\beta {\rho_{B}\mu }_{B}I}$,$\frac{C-2H}{\beta \rho_{A}\mu_{A}I}$). The location of *T* point is influenced by the complementarity of EHR resources (β), hospital’s willingness ($\rho_{i}$) and ability to use HER ($\mu_{i}$), the average income of unit EHR resources (*I*), unit EHR resource sharing cost (*C*), and unit resource reward and punishment (*H*). The influence mechanism of the relevant parameters is analyzed below. Where the area of the FTGK region can be expressed as follows [26]:

$S_{FTGK}=1-\frac{1}{2}\left(x^{*}+y^{*}\right)=1-\frac{1}{2}\left(\frac{C-2H}{\beta \rho_{B}\mu_{B}I}+\frac{C-2H}{\beta \rho_{A}\mu_{A}I}\right)$(11)

#### The Influencing Mechanism of Original Revenue $R_{i}$ and EHR Stock $r_{i}$

**Figure 2.**
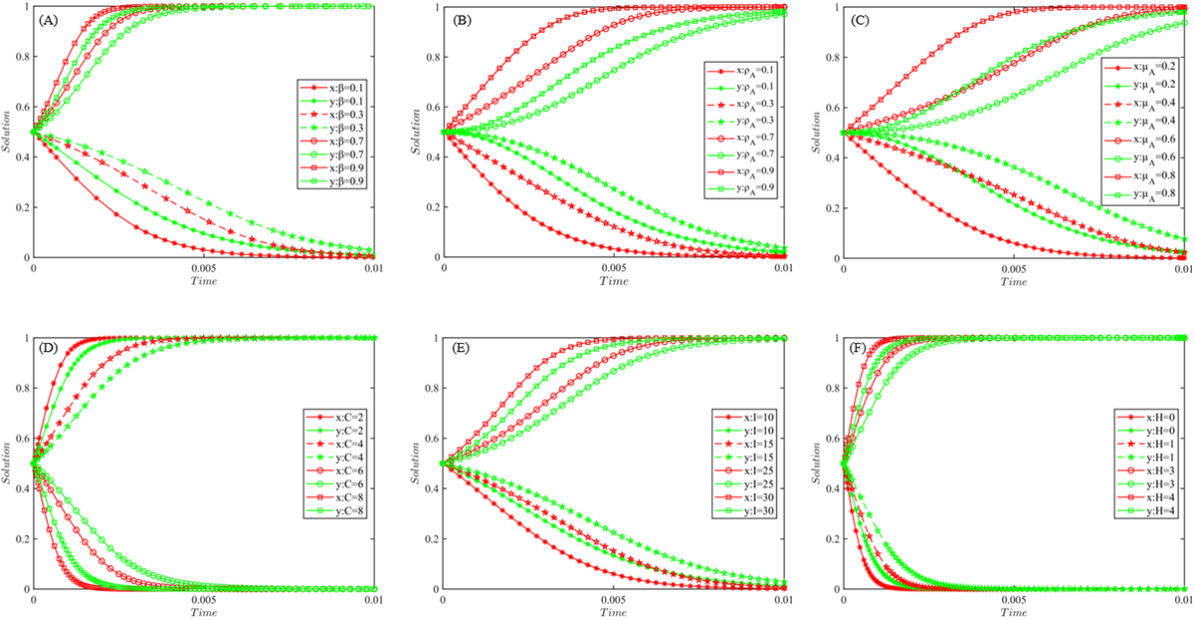
Simulation results of influence effects based on MATLAB. (A) Effect of *β* on the evolutionary game system, (B) effect of $\rho_{i}$ on the evolutionary game system, (C) effect of $\mu_{i}$ on the evolutionary game system, (D) effect of C on the evolutionary game system, (E) effect of I on the evolutionary game system, and (F) effect of *H* on the evolutionary game system.

The location of the saddle point T in Figure 2 determines the likelihood of the evolutionary direction of the game system. The position of saddle point T depends on the combined effect of multiple factors. However, according to the model solution results equation (11), it can be seen that these factors do not include the original revenue $R_{i}$ and the stock of EHRs $r_{i}$. This indicates that hospitals’ original revenue and EHR stock have no substantial effect on the evolutionary direction of the gaming system. One reason is that the superior hospitals we conceptualize are not equivalent to large hospitals, and subordinate hospitals are not equivalent to small hospitals either. More importantly, incentives, penalties, and costs are calculated on the basis of “EHR resources per unit,” not on the basis of the hospital’s original revenue and total EHR.

#### The Influencing Mechanism of EHR Resource Complementarity (*β*)

Let $S_{FTGK}$take the derivative of β, we can get

$\frac{\partial S_{FTGK}}{\partial \beta }=\frac{1}{2}\left(\frac{C-2H}{\beta^{2}{\rho_{B}\mu }_{B}I}+\frac{C-2H}{\beta^{2}\rho_{A}\mu_{A}I}\right)$(12)

Since equation (12) is always greater than zero, it means that $S_{FTGK}$ is an increasing function of the EHR resource complementarity (*β*). The area of the region $S_{FTGK}$ in the upper right of the polyline ETG increases with *β*. With the increase of resource complementarity, the willingness of participating institutions of medical consortiums to use EHR will increase, which will help promote close cooperation between superior and subordinate hospitals, and thus promote the creation of integrated medical value by medical consortiums. On the contrary, if the EHR complementarity is low, then hospitals at all levels will be more willing to use their own generated EHR. Even if it is mandatory under the policy, hospitals are likely to only share their own EHR and will not take the initiative to use the EHR shared by other hospitals. This would go against the original intention and social expectations of EHR integration. The impact path of the EHR complementarity (*β*) on the co-opetition strategy of the superior and subordinate hospitals is reflected in the phase diagram, that is, as *β* increases, the saddle point T will keep moving toward the *O* (0, 0), which makes the upper right area of ETG keep increasing, which keeps increasing the probability of the superior and subordinate hospitals choosing the {cooperation, cooperation} strategy.

It is important to note that because $\beta {{,\rho }_{i},\mu }_{i}and$ *I* have similar mathematical properties, meaning that these 4 parameters have the same mechanism of action on the influence of the co-opetition strategies of the superior and subordinate hospitals. That is to say, with the increase of the value of the 4 parameters of EHR resource complementarity (*β*), hospital’s willingness ($\rho_{i}$) and ability ($\mu_{i}$) to use EHR, the average income of unit EHR resources (*I*), the probability of the superior and subordinate hospitals choosing the {cooperation, cooperation} strategy will increase, and then the game system will evolve to the {1, 1} stable point.

#### The Influencing Mechanism of Unit EHR Resource Sharing Cost (*C*)

Let $S_{FTGK}$take the derivative of C, we can get

$\frac{\partial S_{FTGK}}{\partial \beta }=-\frac{1}{2}\left(\frac{1}{\beta {\rho_{B}\mu }_{B}I}+\frac{1}{\beta \rho_{A}\mu_{A}I}\right)$(13)

Because equation (3) is constantly less than 0, meaning that $S_{FTGK}$ is a decreasing function of the unit resource sharing cost *C*, the area of the region $S_{FTGK}$ at the top right of the polyline ETG decreases with the increase of *C*. With the increase of unit resource sharing cost, the willingness of participating institutions of medical consortiums to use EHR will diminish, indicating that the higher the unit resource sharing cost *C*, the lower the willingness and probability of hospitals to participate in the EHR integration. On the contrary, if the unit resource sharing cost *C* is low, the motivation of hospitals at all levels to share and use EHRs will increase, which is a strong impetus to achieve the goal of EHR integration. The impact path of unit resource sharing cost *C* is reflected in the phase diagram, that is, with the increase of *C*, the saddle point *T* will continue to move to the point *K*, making the area of the upper right of the ETG continue to decrease, weakening the motivation of the superior and subordinate hospitals’ choice of {cooperation, cooperation} strategy; With the decrease of *C*, the saddle point *T* will keep moving toward the point *O*, making the upper right area of ETG keep increasing, keep increasing the probability of the superior and subordinate hospitals choosing the {cooperation, cooperation} strategy.

#### The Influencing Mechanism of Unit EHR Resource Reward and Punishment Intensity (*H*)

Let $S_{FTGK}$take the derivative of H, we can get

$\frac{\partial S_{FTGK}}{\partial \beta }=\frac{1}{\beta {\rho_{B}\mu }_{B}I}+\frac{1}{\beta \rho_{A}\mu_{A}I}$(14)

Since equation (14) is strictly positive, $S_{FTGK}$ becomes a monotonically increasing function of H, indicating that the area of region $S_{FTGK}$ located in the upper-right quadrant relative to the polyline ETG grows with H. With the increase of the unit resource reward and punishment, the probability of using EHR in participating institutions of medical consortiums will keep increasing, indicating that the greater the unit resource reward and punishment intensity *H* the greater the motivation of hospitals at all levels to participate in the EHR integration. On the contrary, if the reward and punishment of unit resources intensity *H* is low, the enthusiasm of hospitals at all levels to share and use EHR will be weakened, which will hinder the realization of the goal of EHR integration to a certain extent. The impact path of *H* on the co-opetition relationship between the superior and subordinate hospitals is reflected in the phase diagram, that is, with the increase of *H*, the saddle point *T* will continue to move toward the point *O*, making the upper right area of the ETG continue to increase, thus increasing the probability of the superior and subordinate hospitals to choose the {cooperation, cooperation} strategy; with the decrease of *H*, the saddle point *T* will continue to move toward the point *K*, making the upper right area of the ETG continue to decrease, and the probability of hospitals choosing the {competition, competition} strategy will increase.

### MATLAB-Based Simulation of Influence Effects

In order to more intuitively describe the influence effect, and to verify the reasonableness and reliability of the analysis of the influence mechanism of the key factors, MATLAB R2018b was used to carry out numerical simulation and analysis of the differential equation (equation 9). When a parameter was simulated, other parameters ware taken as fixed values. The initial values of each parameter are set as follows: $\;r_{A}=800,r_{B}=500,\beta =0.4,\rho_{i}=0.5,\mu_{i}=0.5,I=20,H=2,C=5$, *x* and *y* are taken as 0.5. The simulation results are shown in Figure 2.

### Effect of EHR Resource Complementarity (*β*)

From Figure 2A, the lower the EHR resource complementarity (*β*), the more hospitals tend to choose the competitive strategy. With the increasing complementarity of EHR resources, the strategy choice of superior and subordinate hospitals gradually shifts from competition to cooperation. And after the critical state, the evolutionary game system converges to {cooperative, cooperative} stable strategy at a faster speed. Complementarity refers to the nonoverlapping content of EHRs owned by each of the superior and subordinate hospitals. The more frequent the “up and down referrals” are, the greater the nonoverlap of EHR contents. Therefore, the medical consortiums should actively involve different types of hospitals in the region, and continuously improve the complementarity of EHR resources between superior and subordinate hospitals, while expanding the patient’s right to choose medical treatment.

### Effect of Hospitals’ Willingness ($\rho_{i}$) and Ability ($\mu_{i}$) to Use EHR

From Figure 2B and (C), it can be seen that the willingness and ability to use EHR of superior and subordinate hospitals have similar effects on the evolution of the evolutionary game system. With the increase of hospitals’ willingness or ability to use EHR, the probability of dynamic game system tending to {cooperation, cooperation} state will keep increasing, and vice versa. Both the willingness and ability to use the EHR are prerequisites for creating benefits after EHR integration. The stronger the willingness and ability, the stronger the hospital’s perception of use, the more confident it will be in the expected benefits, and the more motivated it will be to participate in EHR integration.

### Effect of Unit EHR Shared Cost (*C*)

As can be seen from Figure 2D, when the other parameters are taken as fixed values, the probability of choosing the cooperation strategy for superior and subordinate hospitals gradually decreases as the unit EHR sharing cost *C* keeps increasing. When the unit EHR sharing cost *C* is less than the critical value, the probability of the superior and subordinate hospitals chooses to actively cooperate increases continuously with the decrease of *C*. At present, the technical standards and content formats of EHR, EMR and other systems used by different hospitals are likely to be different. To achieve EHR integration, hospitals may need to revamp system interfaces, format content, optimize management practices, and even staff more technical personnel. The increased difficulty of integration will lead to higher integration costs, which will objectively reduce the enthusiasm of hospitals to implement the integration of EHR, and ultimately lead to the evolution of the game system towards a stable strategy of competition.

### Effect of Average Revenue of Unit EHR (*I*)

From Figure 2E, it can be seen that the evolutionary game system gradually converges to the {cooperative, cooperative} state as the average revenue of unit EHR (*I*) keeps increasing, when the other parameters are taken constant. When the average revenue of unit EHR (*I*) is below a critical value, both superior and subordinate hospitals choose a competitive strategy to ensure that their respective interests are maximized. Both the unit EHR sharing cost (*C*) and the unit EHR average benefit (*I*) are crucial to ensure the sustainable development and usage of EHR, and they have a decisive impact on the hospital’s revenue as well as rewards and penalties. Therefore, while promoting the EHR integration, medical consortiums must continuously develop and expand the channels for creating value and revenue, and adopt advanced technologies and measures to gradually reduce sharing costs.

### Effect of Rewards and Punishments of Unit EHR (*H*)

It can be seen from Figure 2F that when the other parameters take fixed values, the probability that both sides of the game choose to cooperate gradually increases with the increase of the unit EHR rewards and punishments (*H*). In order to promote hospitals to actively share and use EHR, and to avoid opportunistic behaviors such as “free-riding,” reward and punishment mechanisms are often established to protect the legitimate benefits of superior and subordinate hospitals. Compared with the unit EHR resource sharing cost (*C*) and the average benefit of unit EHR (*I*), the reward and punishment intensity (*H*) is the easiest to regulate and control. The evolutionary game system is more sensitive to *H*. It means that the strength of rewards and punishments will play a more obvious role in promoting the medical consortiums to achieve the goal of EHR integration.

## Discussion

### Principal Findings

To make both the superior and subordinate hospitals *A* and *B* choose the cooperation strategy and promote the smooth implementation of EHR integration in the medical consortiums, it is necessary to ensure that the initial probability of both sides of the game to choose the cooperation strategy is within the FTGK region (as shown in Figure 2). At this time, the probability requirements for the superior and subordinate hospitals *A* and *B* to choose cooperation are as follows:

$\;x>x^{*}=\frac{C-2H}{\beta \rho_{B}\mu_{B}I}$；$y>y^{*}=\frac{C-2H}{\beta \rho_{A}\mu_{A}I}$ (15)

The complementarity of EHR resources, hospitals’ willingness and ability to use EHRs, and the average benefit of unit EHRs have a positive effect on the realization of EHR integration in medical consortiums. The input cost of unit EHR resource has a negative hindering effect on achieving EHR integration. The original revenue of the superior and subordinate hospitals *A* and *B*, and the stock of EHRs have no effect on the evolutionary direction of the game system. In other words, whether it is a superior hospital or a subordinate hospital, no matter how much or how little EHR that can be shared and used, there is no substantial impact on achieving the goal of the EHR integration in medical consortiums.

Designing a reasonable reward and punishment mechanism is great significance to promote medical consortiums to realize the EHR integration, especially in the initial stage. The integration progress can be accelerated if the rewards and punishments are more than half of the sharing cost. In the initial stage, rewards and punishments can be appropriately increased to speed up the progress of EHR integration. The goal of EHR integration can also be achieved when the rewards and punishments are less than half the sharing cost under the condition of $\;\beta \rho_{i}\mu_{i}I>C-2H$. Therefore, with the deepening of the EHR integration, the increasing willingness and ability of hospitals to use EHR, and the increasing efficiency of sharing EHR, government subsidies can be appropriately reduced or even eliminated according to the actual situation, thus reducing financial pressure.

### Management Insights

To promote the enthusiasm of superior and subordinate hospitals to share and use EHR, and increase the probability of choosing cooperation strategies, the following aspects need to be emphasized. First, the medical consortiums should actively involve different levels and various types of medical institutions (general hospitals, maternal and child health care centers, specialized hospitals, rehabilitation hospitals, etc.) to improve the complementarity of EHR resources. Second, we should continuously improve the subjective willingness and ability of hospitals to use EHR through training, assistance, support, medical resources sinking and other ways. Third, the government should establish a reward and punishment system, optimize the operation mechanism and supervision mechanism of the medical consortiums, supervise and punish opportunistic behaviors such as “free-riding,” and ensure that the goals of the hospital are consistent with those of the medical consortiums. Fourth, hospitals should continuously strengthen the construction of information infrastructure, actively improve the technical standards, content standards and sharing standards for the EHR construction [27], further improve the benefits of EHR, and reduce the investment cost of EHR sharing and use.

The decision-making groups are the actual game players of the hospital. The hospital’s willingness to use EHR is fundamentally determined by the cognitive level of the decision-making groups. Therefore, the choice of hospital decision makers also has an important influence on the promotion of EHR integration. In the appointment of hospital decision makers, especially the head of the appointment, the cognitive pattern, medical values, social responsibility, dedication and commitment of the appointment candidate should be fully considered. At the same time, medical consortiums should adopt a wide publicity and mobilization, design and announce a reward and punishment system at the early stage of embarking on the integration of EHR to improve the motivation of hospital decision makers to adopt the cooperation strategy. Ensure that the policies and measures can receive positive response and support from the hospital’s decision-making collective, to lay a solid foundation for the realization of the EHR integration.

Hospitals participating in the same consortium should have the same institutional constraints, both at superior hospitals and subordinate hospitals. There should be no differentiation in the system based on the size of the hospital. More importantly, in order to promote the active participation of both superior hospitals and subordinate hospitals in the integration of EHR, the design of incentive and penalty mechanisms and cost accounting should be based on the “unit EHR resources,” rather than on the original revenue of the hospital and the total amount of EHR.

### Research Limitations

In this paper, we take superior and subordinate hospitals within the medical consortium as the research object to explore the evolution mechanism of co-opetition strategies in the EHR integration. Therefore, we adopted a 2-party evolutionary game without considering the game scenario involving more stakeholders, such as the government, integrators, and patients. In addition, due to the lack of real data in practice, only the MATLAB simulation method was used to verify the model rationality, while failing to use empirical methods to test the accuracy of the model. With the continuous deepening of practice, it is necessary to continuously collect relevant data in the future and use empirical methods to further test the scientific validity of research findings.

## Abbreviations

EHR: electronic health record
